# Galectins from *Onchocerca ochengi* and *O. volvulus* and their immune recognition by Wistar rats, *Gudali zebu* cattle and human hosts

**DOI:** 10.1186/s12866-020-02064-3

**Published:** 2021-01-06

**Authors:** Ngwafu Nancy Ngwasiri, Norbert W. Brattig, Dieudonné Ndjonka, Eva Liebau, Archile Paguem, Dustin Leusder, Manchang Tanyi Kingsley, Albert Eisenbarth, Alfons Renz, Achukwi Mbunkah Daniel

**Affiliations:** 1 University of Ngaoundéré, Ngaoundéré, Cameroon; 2 Department Molecular Medicine, Bernhard Nocht Institute of Tropical Medicine, Hamburg, Germany; 3 University of Muenster, Münster, Germany; 4 Department Comparative Zoology, Eberhard Karls University, Institute of Evolution and Ecology, Tübingen, Germany; 5 Department of Veterinary Medicine, University of Buea, Buea, Cameroon; 6 Veterinary Research Laboratory, IRAD Wakwa Regional Centre, Ngaoundéré, Cameroon; 7 Programme Onchocercoses, Station of the University of Tübingen, Ngaoundéré, Cameroon; 8 TOZARD Research Laboratory, P.O. Box 59, Bambili-Tubah, Bamenda, Cameroon

**Keywords:** Recombinant galectin, Somatic extract, *Onchocerca ochengi*, *Onchocerca volvulus*, Excretory-secretory products

## Abstract

**Background:**

During the last two decades research on animal filarial parasites, especially *Onchocerca ochengi,* infecting cattle in savanna areas of Africa revealed that *O. ochengi* as an animal model has biological features that are similar to those of *O. volvulus,* the aetiological agent of human onchocerciasis. There is, however, a paucity of biochemical, immunological and pathological data for *O. ochengi*. Galectins can be generated by parasites and their hosts. They are multifunctional molecules affecting the interaction between filarial parasites and their mammalian hosts including immune responses. This study characterized *O. ochengi * galectin, verified its immunologenicity and established its immune reactivity and that of *Onchocerca volvulus* galectin.

**Results:**

The phylogenetic analysis showed the high degree of identity between the identified *O. ochengi* and the *O. volvulus* galectin-1 (ß-galactoside-binding protein-1) consisting only in one exchange of alanine for serine. *O. ochengi* galectin induced IgG antibodies during 28 days after immunization of Wistar rats. IgG from *O. ochengi*-infected cattle and *O. volvulus*-infected humans cross-reacted with the corresponding galectins. Under the applied experimental conditions in a cell proliferation test, *O. ochengi* galectin failed to significantly stimulate peripheral blood mononuclear cells (PBMCs) from *O. ochengi*-infected cattle, regardless of their parasite load.

**Conclusion:**

An *O. ochengi* galectin gene was identified and the recombinantly expressed protein was immunogenic. IgG from *Onchocerca*-infected humans and cattle showed similar cross-reaction with both respective galectins. The present findings reflect the phylogenetic relationship between the two parasites and endorse the appropriateness of the cattle *O. ochengi* model for *O. volvulus* infection research.

**Supplementary Information:**

The online version contains supplementary material available at 10.1186/s12866-020-02064-3.

## Background

Human onchocerciasis (river blindness) caused by *Onchocerca volvulus* is endemic in 30 countries of sub-Saharan Africa and Yemen. Onchocerciasis presently affects an estimated 17 million people worldwide and 99% of the infected persons live in sub-Saharan Africa [1]. Studies on the biology of *O. volvulus* have been limited due to the lack of appropriate animal models [2]. However, during the last three decades, the natural infection of cattle by *O. ochengi* has been used as an animal model due to its close phylogenetic relation to *O. volvulus*, with which it shares the same vector species, the black-fly *Simulium damnosum s.l.* [3–7]. Cattle infected with *O. ochengi* have nodules that can be counted by palpation of the skin or be removed for immunological or chemotherapeutic analysis [8–10].

The clinical signs of onchocerciasis in man vary from mild to hyper-reactive forms. In both, humans and cattle, some individuals are nearly or completely free from the parasite despite living in highly endemic areas. These individuals are usually considered as being resistant to infection and called ‘putatively immune individuals’ (PI) [9, 11]. Parasitic nematodes release products into their hosts, which enable them to invade, develop and persist by modulating the host’s immune response [12–14]. Protease inhibitors, antioxidants and orthologues of host cytokines and lectins [15, 16] contribute to the establishment of infection in various ways: They are involved in vital biological processes such as proliferation, cells differentiation or immune cell responses. Such molecules can be collected as excretory-secretory products (ESPs) from worms kept in vitro [17, 18] and constitute potential targets for the development of new control intervention strategies [19, 20]. Among such products are proteins, which specifically bind to ß-galactoside glycan [18]. These are generally referred to as galectins and they are known to have immunomodulatory functions under various conditions [18, 21, 22]. Therefore, galectins have been considered useful targets for the development of an anti-parasitic vaccine or for therapy [23, 24]. They are capable of down-regulating protective Th1 immunity, enabling survival of the parasite within the host [23, 13]. Also, they have been shown to participate in the regulation of both innate and adaptive immunity [25–27]. Although galectins lack a classical secretion signal peptide, they localize to both intracellular and extracellular compartments. Galectins function through protein-protein or protein-glycan interactions [24–26]. Galectins secreted by the parasite can bind to its cuticula and initiate immune responses of the mammalian host [25, 28, 29]. Secreted by all stages of the worms, galectins may be essential for survival of *Onchocerca* filariae in their definitive hosts [30].

In the present study, an *O. ochengi* galectin was recombinantly produced and used to study its immunogenic activity through evaluation of antibody production in immunized Wistar rats. *O. ochengi* and *O. volvulus* galectins were used to test for cross-reactivity in sera from *Onchocerca* spp*.* infected cattle and human subjects. PBMCs from *O. ochengi-*infected cattle with strikingly different parasite loads were stimulated in vitro with recombinant *O. ochengi* galectin for immune modulation evaluation.

## Results

### Gene expression and purification of *O. ochengi* galectin

**Fig. 1:**
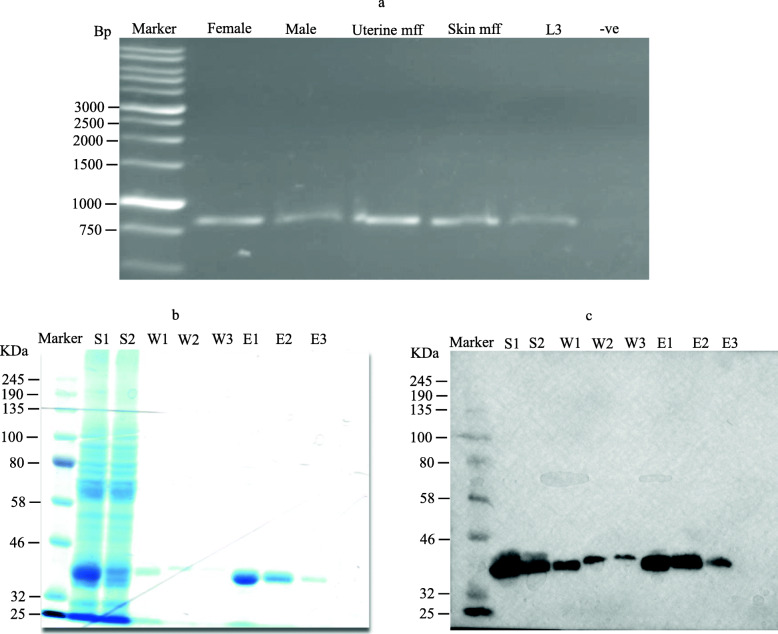
Amplification products of *O. ochengi* cDNA transcribed from RNA isolated from *O. ochengi* worm stages. **a**: Amplification products of *O. ochengi* cDNA transcribed from RNA which was isolated from *O. ochengi* adult female and male worms, uterine and skin microfilariae, and L3 as templates on 1% agarose gel. The length of the cDNA is about 850 bp. **b**: Course of affinity chromatography purification of *O. ochengi* recombinant galectin using Ni-NTA resin and selected fractions ran on a 12% SDS-PAGE gel. **c**: corresponding western blot using anti-His antibody. The lanes show: Marker: standard proteins, lane S1: supernatant before binding to beads, lane S2: supernatant after binding to beads, lanes W1, W2, W3: wash fractions, lanes E1, E2, E3: eluted fractions of recombinant *O. ochengi* galectin. E3 shows a decrease in the amount of recombinant *O. ochengi* galectin present in sample. Three separate elutions (E1, E2 and E3) separated by an interval of 10 min were undertaken to maximize the quantity of eluted proteins

RNA was isolated from adult *O. ochengi* female and male worms, uterine and skin microfilariae (mff) and infective third-stage larvae (L3). First-strand complementary DNA was synthesized and used as a template for the amplification of the target sequence. Here a PCR product of approximately 850 bp was amplified from all worm stages (Fig. 1a).

The amplified *O. ochengi* galectin cDNA from adult female was successfully isolated and cloned in the histidine-tagged pJC40 expression vector. The recombinant protein was purified using Ni-NTA affinity chromatography and samples of each purification step were analyzed by SDS-PAGE (Fig. 1b). The *O. ochengi* galectin has the expected molecular weight of approximately 34 kDa (Fig. 1b and c). Recombinant protein expression was confirmed by western blotting using anti-His antibody (Fig. 1c).

### Sequence analysis and phylogenetic tree

**Fig. 2:**
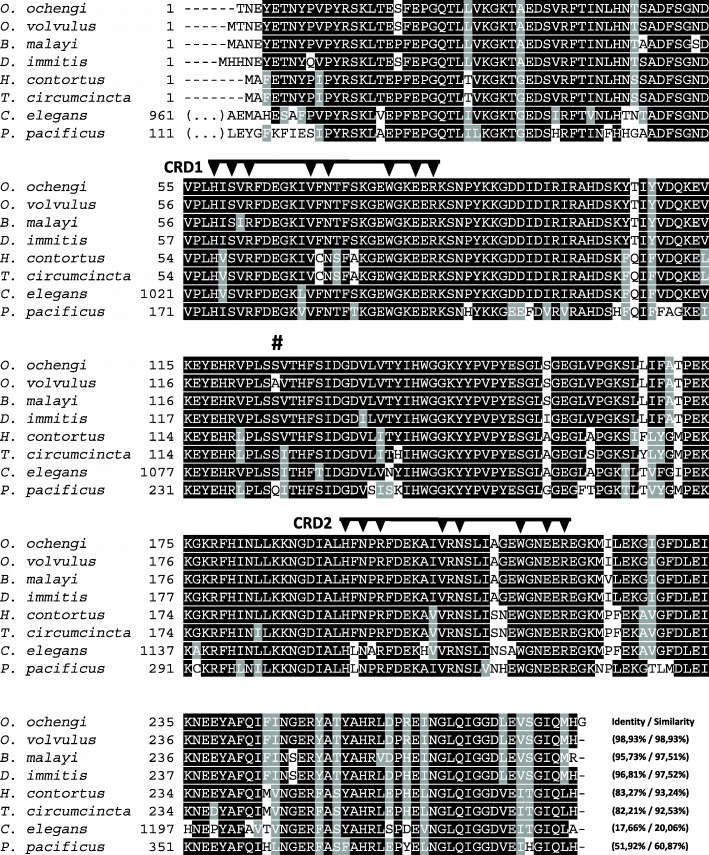
Alignment of amino acid sequences of *O. ochengi* galectin compared to other nematode galectins. Alignment of amino acid sequences of *O. ochengi* galectin (O*. och* Gal) compared to galectins from *O. volvulu*s (AAA20541.1), *Brugia malayi* (XP_001896448.1), *Dirofilaria immitis* (AAF37720.1), *Haemonchus contortus* (AAD11972.1), *Teladorsagia circumcincta* (AAC47546.1), *Caenorhabditis elegans* (NP_496165.3) and *Pristionchus pacificus* (PDM78135.1). Identical amino acids are highlighted in black while similar amino acids are highlighted in grey. Dashes indicate gaps in sequences. The carbohydrate recognition domains (CRD) are marked with arrows [32]. The # indicates the single amino acid exchange between *O. ochengi* and *O. volvulus*. In brackets, the amino acid identity and similarity is given compared to the *O. ochengi* sequence

**Fig. 3:**
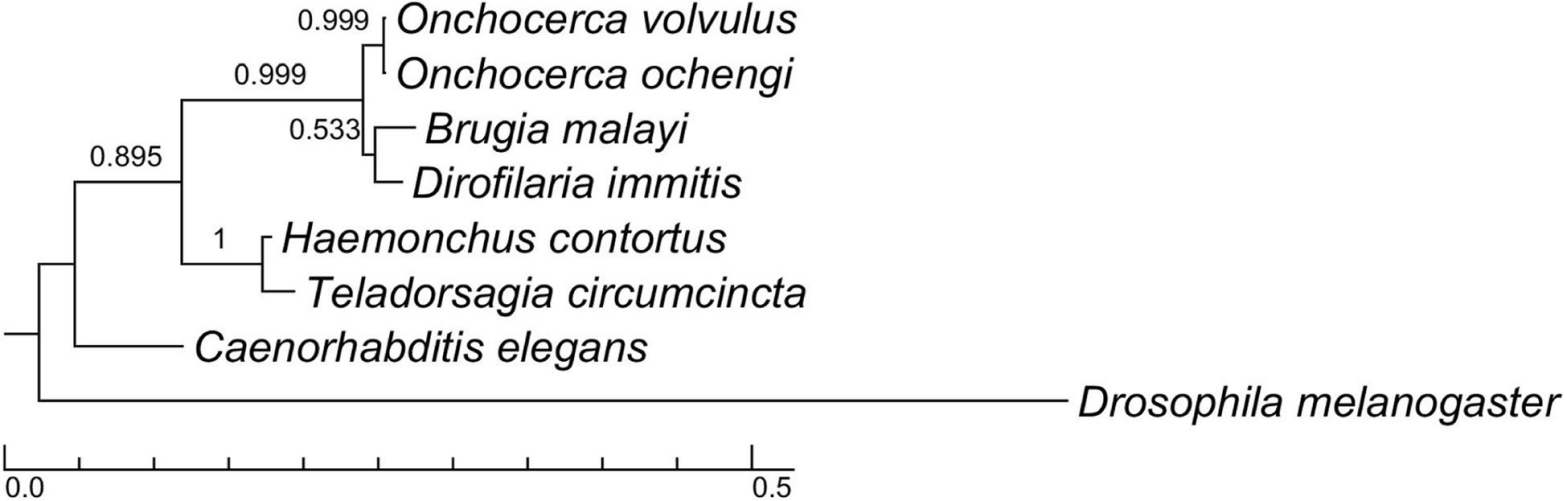
Phylogenetic consensus tree from the galectin sequences of different nematodes. Phylogenetic consensus tree obtained from the galectin sequences of nematodes (Fig. 2). *Drosophila melanogaster* galectin (AAL87743.1) was used as an outgroup. The tree was calculated through the Bayesian inference model with a Markov chain Monte Carlo method using the following parameters in the program ‘MrBayes‘: 5.000.000 generations were calculated based on the GTR + I + G substitution model. Print frequency was set to 100 while sample frequency was set to 1000. Branch length indicates the relative evolutionary distance. Values on the branches indicate the relative amount of sample trees supporting the consensus tree

Following sequence analysis, the deduced amino acid sequence was shown to be almost identical to the galectin GBP-1(ß-galactoside-binding protein-1) from the sister species *O. volvulus* [30, 31, 33]; with only one amino acid exchange, alanine versus the biochemically connatural serine (hydroxy-alanine). Furthermore, sequence alignment of *O. ochengi* galectin with other nematode galectins revealed a high degree of sequence conservation. Based on the presence of predicted carbohydrate recognition domains (CRDs), it is a tandem-repeat type galectin (Fig. 2). The sequence analysis of the CRDs shows that the investigated *O. ochengi* galectin is homologous to the OvGPB1 and the Lec-2 from *Brugia malayi*. The calculated evolutionary distance between galectin family members of various nematodes demonstrates the close relationship between galectins from filarial parasites (Fig. 3).

### Hemagglutination activity

**Fig. 4:**
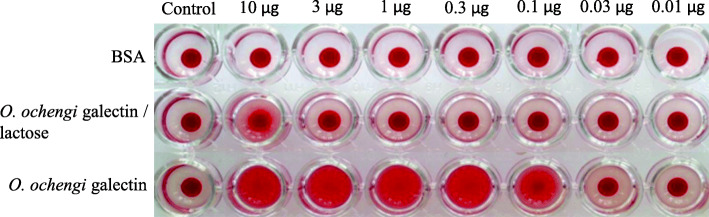
Hemagglutination activity of recombinant *O. ochengi* galectin to human group B erythrocytes. Lane 1: negative control with 35 μl of PBS and 35 μl of 2% erythrocytes suspension; Lane 2: 10 μg of protein in 35 μl PBS and 35 μl of 2% erythrocytes suspension; Lane 3–8: 10 μg of protein in 35 μl PBS serially diluted (3-fold) in PBS and 35 μl of 2% erythrocytes suspension to each diluted sample. 200 mM of lactose was used per well for the hemagglutination assay of the blood B type erythrocytes. Bovine serum albumin (BSA) negative control for agglutination

The binding of the galectins to (β1–4)-linked N-acetyl-lactosamine (LacNAc) residues was verified by hemagglutination of the galectin to human type B erythrocytes, which is characterized by surface Gal(ß1–4) LacNAc residues. The recombinant *O. ochengi* galectin showed hemagglutination activity of the type B erythrocytes indicated by the fuzzy spread of erythrocytes to a concentration as low as 0.01 μg/well. For confirmation of this specific binding, the agglutination of *O. ochengi* galectin was inhibited by lactose (Fig. 4).

### Humoral IgG response of Wistar rats immunized with *O. ochengi* galectin using *O. ochengi* extract and *O. ochengi* galectin as antigens

Recombinantly expressed *O. ochengi* galectin was not recognized by IgG in sera of naïve rat while rats immunized with *O. ochengi* galectin recognized the galectin marginally after 14 days and partially after 28 days (Table 1). *O. ochengi* somatic extracts were strongly recognized by IgG from both immunized and non-immunized rats.

**Table 1:** ELISA antibody titres of sera from rats immunized with *O. ochengi* recombinant galectin GBP-1 and female worm extract at 0, 14 and 28 days post immunization

Antigens	OD, Non-immunized Wistar rat (control)		OD, Median (IQR) Immunized Wistar rats	
	***O. ochengi*** galectin	***O. ochengi*** extract	***O. ochengi*** galectin	***O. ochengi*** extract
D_0_	0	3060	0.0 (0.0)	3466 (2914–4136)
D_14_	0	3139	0 (0–173)	2960 (2724–3532)
D_28_	0	2492	160 (0–2790)	3374 (2795–3831)

Rats (*n* = 3) were immunized with 20 μg of recombinant *O. ochengi* galectin protein on day 0 and a booster dose was given 14 days thereafter. Blood samples were collected before immunization (day 0; D 0), 14 days after immunization (D 14) and 14 days after boosting (D 28). As a negative control, a non-immunized rat was used ( *n*= 1). Sera were analyzed by ELISA for total IgG antibodies with *O. ochengi* recombinant galectin and *O. ochengi* somatic extract as antigens. Mann-Whitney *U* test was used for this analysis. *OD* Optical density (450 nm); *IQR* interquartile range of OD values (indicated in brackets)

### Cross-reactivity of IgG antibodies with *O. ochengi* and *O. volvulus* galectin

**Fig. 5:**
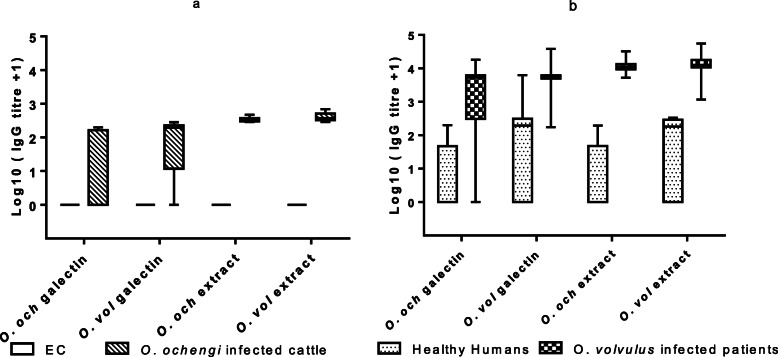
Recognition and cross-reactivity of *O. ochengi* and *O. volvulus* galectins in cattle and human sera. Somatic extracted proteins from these filarial worms were additional tested. **a**: shows the recognition and cross-reactivity of these antigens with sera from *O. ochengi*-infected cattle (*n* = 9) and naïve European cattle (*n* = 2), and **b** indicates these responses in sera from *O. volvulus*-infected humans (*n* = 44) and healthy Europeans (*n* = 12). The figures present median of IgG titres (± IQR quartiles)

Sera of *O. ochengi-*infected cattle (Fig. 5a) and O*. volvulus-*infected humans (Fig. 5b) recognized recombinant *O. ochengi* as well as *O. volvulus* galectins and most strongly reacted with proteins in the respective *Onchocerca* somatic extract (Additional file 1). IgG in sera from *O. volvulus*-infected humans strongly reacted with *O. volvulus* galectin as well as *O. ochengi* galectin showing the robust IgG cross-reactivity (Fig. 5b).

### Lack of a cell proliferation response after in vitro exposure of PBMCs from *O. ochengi*-infected cattle to *O. ochengi* galectin and female worm extract

In order to analyze the capability of the *O. ochengi* galectin to induce cellular immune response in *O. ochengi*-infected cattle, we exposed PBMCs from cattle with high and low nodule and mff loads to the galectin and to female worm extracted proteins. The polyclonal cell activating lectin ConA was used as a positive control. After a culture period of 3 days we observed a strong lymphocyte proliferation of PBMCs exposed to ConA (median optical density between 1.056–1.275). Irrespective of the parasite loads, there was no significant cell proliferation induced by *O. ochengi* recombinant galectin or female worm extract (Table 2).

**Table 2:** Peripheral blood mononuclear cells from cattle with different parasitic loads lack response to *O. ochengi* galectin and *O. ochengi* worm extract

Antigens	OD of animals with low/high nodules load (NL)		OD of animals with low/high Mff density (MD)	
	Median (IQR)		Median (IQR)	
	Low	High	Low	High
Control (culture medium)	0.119 (0.096–0.123)	0.125 (0.079–0.133)	0.119 (0.101–0.174)	0.084 (0.079–0.126)
*O. ochengi* galectin	0.133 (0.097–0.151)	0.133 (0.095–0.166)	0.151 (0.105–0.227)	0.099 (0.092–0.146)
*O. ochengi* extract	0.099 (0.083–0.118)	0.098 (0.083–0.127)	0.104 (0.083–0.154)	0.085 (0.082–0.109)
ConA.	1.275 (0.618–1.570)	1.364 (0.279–2.094	1.289 (0.618–1.531)	1.056 (0.279–1.725)
Control: low vs high (NL or MD)	*P* = 0.754		*P* = 0.251	
*O. ochengi* galectin: low vs high (NL or MD)	*P* = 0.675		*P* = 0.402	
*O. ochengi* extract: low vs high (NL or MD)	*P* = 0.917		P = 0.251	
ConA: low vs high (NL or MD)	*P* = 0.0754		P = 0.754	

Mann-Whitney *U test* analysis of the proliferation of PBMCs from cattle using cattle with low and high parasite loads (low parasite load: 0–16 nodules, 0 mff/mg,of skin, high parasite load: 103–920 nodules, 8–21 mff/mg of skin), *n* = 5 per group. PBMCS were exposed for 3 days to *O. ochengi* galectin, *O. ochengi* extract from females worms and to polyclonal activator ConA as positive control. Applying the MTT proliferation assay the absorbance of the MTT was measured as optical density (OD). *IQR* interquartile range of OD indicated in brackets

## Discussion

Here we report the identification, cloning and first functional characterization of an *O. ochengi* galectin. Amino acid sequence analysis revealed only one exchange of a very related amino acid (alanine vs. hydroxy-alanine, serine) compared to the galectin GBP-1 from its sister species *O. volvulus* [30]. Furthermore, sequence analysis indicates that the *O. ochengi* protein is the orthologue of *Brugia malayi* BmLec-2.

The tandem repeat galectin has two essential CRDs that mediate the functional activity of the recombinant protein. This is confirmed by the hemagglutination activity against human group B erythrocytes expressing β (1–4)-linked N-acetyl-lactosamine residues. Galectins have been identified and characterized in a myriad of pathogens [25, 28], particularly in protozoa like *Leishmania* [33] and in helminths including numerous filariae like *O. volvulus* [30], *Brugia malayi* [20, 32] and *Dirofilaria immitis* [34]. Furthermore, galectins occur in the nematode genera such as *Toxascaris spp* [35] *Haemonchus spp* [36] and *Trichinella spp* [37]. Galectins were also reported from trematodes like *Fasciola spp* [24].

Ditgen et al. [26] characterized two galectins of the intestinal parasite *Strongyloides* and also demonstrated hemagglutination activity with human group B erythrocytes. Furthermore, galectin from *Strongyloides ratti* was found to interact with intestinal epithelial cells and immune cells. Here we report evidence that the recombinant *O. ochengi* galectin investigated in this study is a tandem repeat galectin comprising two CRDs tightly binding to N-acetyl-lactosamine determinants on blood group B erythrocytes. Lactose represents a ligand for galectins which also prevents the interaction between galectin and blood group A determinant [24, 38]. Thus, the hemagglutination assay verified the nature of galectin as characteristically binding proteins to (β1–4)-linked N-acetyl-lactosamine (LacNAc). The in vivo functional activities of the galectin, however, remain unexplained. Optionally, the *O. ochengi* galectin may bind to glycan structures on host cells directly adjacent to the filaria in the skin or onchocercoma which putatively mediate their interaction with the tissue parasite.

The recombinant *O. ochengi* galectin induced a time-dependent IgG antibody response in immunized rats, indicating that the defence system of the mammal is activated and can block the physiological functionality of galectin by binding to host’s cell N-acetyl-lactosamine determinants with subsequent agglutination or blocking of their physiological function and thus may be operative in the defence mechanism. The IgG binding with galectin was analysed by ELISA.

Unexpectedly, sera from the rats irrespective of the time point during the immunization process showed high IgG reactivity with proteins in the *O. ochengi* extract*.* Such antibody responses appear to indicate the rats’ immune system had been exposed earlier to numerous proteins which may cross-react with the extracted proteins from the filariae. In a report of Younis et al. [39], rats immunized with the nematode protein *Strongyloides ratti* HSP-17 likewise produced a time-dependent IgG antibody response. Of interest, IgG antibodies against the nematode protein Sr-HSP-17could also be demonstrated after infection of naïve rats with the parasite *S. ratti* indicating a release of this nematode protein and its exposure to the rat immune system. Furthermore, humans infected with *Strongyloides stercoralis* also reacted with the Sr*-*HSP-17 indicating strong cross-reactivity between the two *Strongyloides* species.

The antibody response detected in sera from *Onchocerca*-infected cattle was generally low for the tested antigens, i.e. to recombinant galectin from both worms (*O. ochengi* and *O. volvulus*) and higher for proteins in the worm extract. Both species produced a robust antibody response that readily recognized the *O. volvulus* and *O. ochengi* galectins. A direct comparison of the strength of the antibody activity in cattle and humans is not indicated by the data. In the present study, PBMCs from cattle with low or high *O. ochengi* burden - based on nodule loads and mff densities in the skin - did not show any significant in vitro proliferation in the presence of *O. ochengi* galectin nor to proteins in *O. ochengi* extracts, but proliferated when exposed to the polyclonal stimulant lectin ConA as positive control (Table 2). Reasons may be a too short culture period of the exposed PBMCs or a too low cell culture concentration.

Similarly, other studies reported a poor proliferative response in cells stimulated with both *O. volvulus* and *O. gutturosa*-extracted antigens [40]. An absence of PBMCs proliferation in vitro in the presence of filarial antigens was also found in microfilaremic individuals [41], and a similar response has been found with *O. ochengi* whole worm extracts [6]. PBMCs from hookworm-infected patients showed a reduction in cell proliferation after stimulation with various excretory/secretory proteins of adult worms and infective third-stage larvae [42]. This reduction of the PBMCs proliferative response may result from activated down-modulatory immune mechanisms [17]. Much in contrast, the protein Ov103 promoted the proliferation of splenocytes derived from mice infected with *Litomosoides sigmodontis* and also *L. sigmodontis* extracts promoted cell proliferation in this mouse-model [43]. An absence of proliferative responses was reported for embryonic and microfilarial antigens compared to adult worm antigens in immunized mice experimentally infected with *O. lienalis* [44]. Therefore, given that our crude extract originated from adult *O. ochengi* females, it is possible that the females contained embryonic and microfilarial (uterine) immunomodulating molecules, which might have accounted for the lower degree of proliferation in the protein extract compared to the pure *O. ochengi* galectin protein although the observed difference was not significant (*P* > 0.05). Apparently, the source of parasite antigen used for cell stimulation highly determines the level of proliferation following reports by Mahanty et al. [45]. They demonstrated that antigens from a mixture of adult female and male *Wuchereria bancrofti* worms down-regulated the proliferative cell responses, while cultures with only adult male antigens had no such effect. This may indicate a role of uterine embryonic stages in the down-regulation of proliferative responses as shown for human onchocerciasis [46, 47]. In a similar cell proliferation approach with *O. ochengi*, quantitative differences in lymphocyte proliferation did not reflect the variation in the number of adult worms or mff density in the animals Graham et al. [48]. Their findings, which corroborates with ours, revealed that PBMCs responses to ConA occurred in all animals with proliferative responses exceeding those of parasite antigen-stimulated cultures. Therefore, even though recent studies have suggested that immunosuppression may be mediated by alternatively activated macrophages [49, 50], the pivotal mechanisms behind down-regulation of cellular proliferative responses during helminth infections still remain relatively unsolved.

*Onchocerca ochengi* and *O. volvulus* galectins were shown in the present study to be phylogenetically closely related confirming earlier reports of Xie et al. [51], Wahl et al. [52], Morales-Hojas et al. [53] and Armstrong et al. [54]. The much higher antibody response observed for *O. ochengi* somatic extracts as compared to *O. ochengi* galectin reflect the presence of a myriad of active compounds in the extracts compared to the single galectin protein. Similar observations were made by Brattig [47] and Manchang et al. [43] using OvALT-2, OvNLT-1, Ov7, *O. volvulus* and *O. ochengi* lysate (extracts). Mpagi et al. [55] found the same in onchocerciasis patients. It has to be considered that an *O. volvulus* infection in humans can endure up to 15 years, while the *O. ochengi* infection in cattle exist for about 10 years.

Individuals with longer periods of parasitism and higher *Onchocerca* worm burden generally produced higher antibody responses compared to individuals with shorter duration [43], and this was also seen in cattle infected with *O. ochengi* [9]. Those cattle had higher IgG1 responses after  four years compared to those at one year of infection. Further, persons with a generalized form of onchocerciasis produced lower IgG levels to *O. volvulus* crude worm antigens compared to those with a hyper-reactive form [43].

## Conclusion

*O. ochengi* galectin was identified in all studied worm stages: microfilariae, infective third-stage larvae, adult males and females. A difference in only one amino acid between the studied *O. ochengi* and *O. volvulus* galectins confirmed their phylogenetic relationship referred to as *Onchocerca* sister species. Galectin from cDNA which originated from RNA isolated from adult female *O. ochengi* was cloned and expressed in *Escherichia coli*. The purified protein was comfirmed as galectin binding-specific – i.e. (β1–4)-linked N-acetyl-lactosamine (LacNAc) residues.

The immunogenicity of *O. ochengi* galectin was manifested by increasing recognition of IgG in sera from rats after immunization with the galectin. This result, however, does not indicate that the antibody inhibits galectin function or that the antibody response is protective. Under the applied experimental condition, *O. ochengi* galectin failed to induce the proliferation of lymphocytes from *O. ocheng*i-infected cattle with low and high worm loads. IgG from *Onchocerca*-infected humans and cattle showed similar cross-reaction with both respective galectins. These findings reflect the phylogenetic relationship between the two parasites and endorse the appropriateness of the cattle *O. ochengi* model for *O. volvulus* infection research.

## Methods

### Design, setting and procedures of the study

#### *O. *o*chengi*

Gudali zebu cattle were purchased with funds from the DFG project from three local cattle breeders when they were at most 2 weeks-old calves and taken to the project paddock. This paddock is situated some 15 km south-west of Ngaoundéré in the neighborhood of the village Galim along the banks of river Vina du Sud (07°12′ 05“N, 013°34’ 52”E, 1063 m altitude) where the cattle were exposed to natural transmission of *O. ochengi* by *Simulium* fly bites.

Nearly all the animals acquired *O. ochengi* nodules and produced mff in the skin after 8 to 15 months. Blood collection and other assays on cattle were approved by the Scientific Directorate of the Institute of Agricultural Research for Development, Cameroon. Blood was collected from the jugular vein in vacutainer tubes containing EDTA and centrifuged at 784 x g for 15 min. Sera from 9 cattle at the age of 20 months (5 females and 4 males) were used to test for cross-reactivity. For proliferation studies of PBMCs, 15 cattle (six males and nine females) exposed to natural *S. damnosum* fly bites for 5 years were reclassified according to their infection levels. Thereafter, blood was collected in heparinized tubes from the 5 least infected and 5 most infected from which the buffy coat mononuclear cells were isolated. Cattle used for PBMCs proliferation were classified into two groups of five animals each, based on their *O. ochengi* nodule loads or skin mff density. The first group based on nodule load was made up of cattle with a low nodule load (0–16 nodules) and the second group of cattle with a high nodule load (103–920 nodules). Classification based on *O. ochengi* mff density was made up of 0 mff/mg in the skin (low mff density) and 9–21 mff/mg in the skin (high mff density). Nodules and mff counts were carried out as described by Renz et al. [4] and Achukwi et al. [9]. Briefly, each animal was restrained in a lateral recumbent position and the nodules counted by palpation of both sides of the animal. Three superficial skin biopsies were collected using a sterile scalpel blade along the *Linea alba*: one biopsy posterior to the umbilicus, one mid-way between the umbilicus and the udder/scrotum and the third just anterior to the udder/scrotum. The skin biopsies directly put in RPMI 1640 medium supplemented with 100 U/ml penicillin and 100 μg/ml streptomycin after collection were incubated at 37 °C for at least 5 h and the first count of mff done following morphological identification using a dissecting microscope as described by Wahl et al. [3]. Skin biopsies were again incubated over night at 37 °C in RPMI 1640 medium supplemented with 100 U/ml penicillin and 100 μg/ml streptomycin after which a second identification and count of mff was made. The skin biopsies were weighed after the second identification and count of mff using a torsion balance (mg). Then, the skin biopsies were digested in 0.25% collagenase B over night at 37 °C and a final identification count was conducted. Mff density per mg of skin was obtained from the total number of mff of each worm species counted divided by the skin biopsies weight [4]. Cattle used in this study were not killed, given that only nodule count and skin snip to determine the parasitic load as well as blood collection for immunological assays was necessary.

#### *O. volvulus parasite* and *O. volvulus* galectin

*O. volvulus* female worms and sera from *O. volvulus* infected persons in Ghana [56, 47] were provided by N.W. Brattig at the Bernhard Nocht Institute of Tropical Medicine (BNITM). *O. volvulus* galectin [57] was provided by E. Liebau from the University of Muenster, Germany.

### Extraction and purification of adult worms from nodules

Extractions of adult male and female worms were done as described by Boursou et al. [58] with some modifications. Briefly, skin of cattle udders containing palpable *O. ochengi* nodules were collected from the municipal slaughter house in Ngaoundéré, brought to the Programme Onchocercoses laboratory and thoroughly washed. Individual nodules were dissected and isolated from the skin using a scalpel blade, and put directly in phosphate buffered saline (PBS-pH 7.2). Male *O. ochengi* were collected by dissection of nodules under a binocular microscope and washed three times in sterile PBS supplemented with 100 U/ml penicillin and 100 μg/ml streptomycin. Female *O. ochengi* worms were isolated by digestion of the nodule with 5% collagenase B for 10 to 15 h at 37 °C and cleaned using sterile PBS supplemented with 100 U/ml penicillin and 100 μg/ml streptomycin [59–61].

### Extraction and purification of skin and uterine microfilariae

Extraction of skin and uterine mff were done from *O. ochengi* infected cattle skin and *O. ochengi* adult female worms, respectively. This was done using the methods described by Beytut et al. [62] for skin mff and that of Bianco et al. [63] and Medina-De la Garza et al. [64] for uterine mff with some modifications. Briefly, *O. ochengi* infected cattle skins (with palpable and visible nodules) around the umbilicus (udder/scrotum) were collected from the Ngaoundéré slaughter house from freshly slaughtered cattle. The skins were washed, rapidly sterilized with 10% hypochlorite solution and shaved using a sterile scalpel blade. The shaved skins were sterilized once more with an antiseptic solution (Betadine) for 5 min and wiped with a sterile compress. Skin snips were collected thereafter under the hood, and transferred into sterile PBS containing penicillin/streptomycin at a concentration of 200 IU penicillin/streptomycin (Pen/Strep) in PBS and incubated for 2 h at 37 °C. Thereafter, emerging mff were collected, filtered and transferred into 15 ml falcon tubes.

For uterine mff, the uterus of female worms containing mff extracted from nodules was transferred into sterile PBS containing Pen/Strep (200 IU) under the hood. The open uterus was incubated in Pen/Strep PBS for 1 h at 37 °C to ease the emergence of mff, which were filtered and collected in falcon tubes. Skin and uterine mff were collected in separate tubes and concentrated by centrifuging at 87 **x g** for 5 min and purified by density gradient centrifugation.

Purification of mff was done by a density gradient using Ficoll (MP Biomedical, Santa Ana, CA, USA; density 1.114 g/ml) and Lymphoflot (Biorad, Munich, Germany; density 1.077 g/ml). Briefly, 3 ml of Ficoll was transferred to a 15 ml falcon tube, carefully overlaid by 3 ml of Lymphoflot and finally by 3 ml of the mff preparation. The mixture was centrifuged for 30 min at 87 **x g**. After centrifugation, the preparation formed three phases with the mff accumulating in the middle phase, which was collected and washed four times in Pen/Strep (200 IU) PBS by centrifuging for 5 min at 87 **x** **g **[62].

### Production of *O. ochengi* infective third-stage larvae (L3)

*Simulium damnosum* blood fed flies were caught in individual containers from cattle that were identified to carry *O. ochengi* nodules and mff. The blood fed flies were brought to the Programme Onchocercoses laboratory and fed daily with 10% sucrose solution containing the fungicide Nipagin soaked with sterile cotton balls. They were maintained in an incubation chamber at 28 °C with humidity within the range of 70 to 90%. After 7 days the surviving flies were dissected for L3 recovery. They were placed on a dissecting slide in a drop of PBS (pH 7.4) and the infective L3 dissected out from the head, thorax and abdomen of the flies. All recovered L3 were transferred to a sterile petri dish containing PBS for cDNA production.

### Identification of *O. ochengi* galectin in extracts from *O. ochengi* adult male and female worms, uterine and skin mff

*O. ochengi* extracts from adult male and female worms, uterine and skin mff were used for the identification of *O. ochengi* galectin protein. *O. ochengi* extracts were loaded with 5 μl of 10 x concentrated loading buffer (Biorad, California, USA). Samples with loading buffer were heated at 95 °C for 5 min, placed on ice for 2 min and loaded onto 12% sodium dodecyl sulphate polyacrylamide gels. After electrophoresis, gels were stained with 0.1% Coomassie brilliant blue stain (Carl Roth, Karlsruhe, Germany), destained with acetic acid-methanol solution and washed with distilled water over night after which only parts containing proteins remained visible as blue bands on the gels. The gels were sent to the University of Greifswald where the proteins (including *O. ochengi* galectin) were identified through LC MS/MS mass spectrometry.

### Production of recombinant *O. ochengi* galectin

#### Preparation of *O. ochengi* cDNA

Whole RNA of *O. ochengi* male, female, mff (skin and uterine) and L3 were extracted using the RNeasy Mini kit (Qiagen, Hilden, Germany) including the column DNAse digest. First strand cDNA synthesis was executed with the AMV First Strand cDNA Synthesis kit (New England Biolabs) using 500 ng RNA and random-hexamer primers according to the manufacturer’s instruction.

### Cloning, expression and purification of *O. ochengi* recombinant galectin

*O. ochengi* galectin was amplified by polymerase chain reaction (PCR) from the cDNA of adult *O. ochengi* female worms using the following two oligonucleotide primers:

Sense - O.ochGal2IF: Xho1.

(5′-AGGATCCGAGC***TCGA***GAACCAACGAATATGAAACGAAT-3′)

and antisense – O.ochGal2IF: **Kpn1.**

(5′-TCGAATTCCCATATG***GTAC***CCTAGTGCATCTGAATACCGCT-3′).

PCR was carried out in a total volume of 50 μl under the following conditions: Initial denaturation at 98 °C for 30 s, followed by 32 cycles each consisting of denaturation at 98 °C for10 sec, annealing at 67 °C for 30 s and elongation at 72 °C for 1 min, concluded with a final elongation at 72 °C for 10 min. After purification and digestion of the PCR product with Xho1 and Kpn1 restriction enzymes, the amplified PCR fragment was cloned into the expression vector pJC40 that encodes a cleavable N-terminal histidine tail of 10 residuals which is added to the gene product and allows for purification via metal chelate chromatography [65]. Positive clones were selected and confirmed by Sanger sequencing. *E. coli* strain BL21 (DE3) bacteria (Stratagene, Germany) were transformed with the respective galectin-pJC40 construct and grown in LB broth medium (Carl Roth, Germany) at 37 °C until OD value at 600 nm wave length reached 0.5. Following induction with 1 mM iso-propyl-β-D-thiogalactopyranoside (IPTG), the *E. coli* (bacteria cells) were incubated overnight at 37 °C under constant shaking after which they were harvested by centrifugation and subsequently stored at −20 °C until used. Bacterial cells were re-suspended with lysis buffer (50 mM NaH_2_PO_4_/Na_2_HPO_4_ pH 7.4, 300 mMNaCl, 0.1% TritonX-100, 10 mM imidazole), containing lysozyme (1 mg/ml, Sigma) and incubated for 30 min on ice for cell lysis. Subsequently, the bacteria cells were sonicated five-times for 30 s with 30% amplitude at 30 W (Sonifier 250, Branson). The lysate was cleared by centrifugation at 30,000 **x g** for 30 min at 4 °C. An affinity chromatography purification step was performed by incubating the supernatant with nickel-nitrilotriacetic acid resin (Ni-NTA, GE Healthcare, Germany) that was previously equilibrated with lysis buffer for 2 h at 4 °C on a shaker. The same purification procedure was previously used in our same laboratory for *O. volvulus* galectin as well as lactose affinity chromatography [57] which gave the same results presented here. Non-specifically bound proteins were washed off with extensive amounts of the wash buffer (lysis buffer containing 20 mM imidazole). Galectin was eluted with buffer containing 250 mM imidazole and dialyzed against PBS using a slide-A-lyzer dialysis cassette (Thermo Fisher Scientific). Three separate elutions (E1, E2 and E3) separated by an interval of 10 min were undertaken to maximize the quantity of eluted proteins.

To reduce the bacterial lipopolysaccharide endotoxin (LPS) load of the recombinant protein preparation, 60 μg/ml of polymyxin B was added to the wash and elution buffers. The homogeneity of the recombinant protein was confirmed by SDS-PAGE analysis [66] and the protein concentrated using Amico Ultra Concentrators (Millipore) according to the manufacturer’s manual. Protein concentration was measured using a NanoDrop (Thermo Fisher Scientific) and protein fractions stored at −20 °C until further analysis.

### Confirmation of *O. ochengi* recombinant galectin protein

To confirm the identity of the purified recombinant protein as the N-terminally tagged *O. ochengi* galectin, elution fractions from the Ni-NTA resin step were analyzed by SDS-PAGE in combination with western blot. After separation of the protein fractions on two 12% SDS gels, one gel was stained with Coomassie brilliant blue (Roti®-Blue, Carl Roth, Karlsruhe) and the other one was used for the transfer of the protein onto a 0.45 mm nitrocellulose membrane (Hybond ECL) by the Bio-Rad Trans-blot system (Bio-Rad, Germany) according to the manufacturer’s protocol. PBS was applied as buffer in the system to identify the protein by means of an anti-His antibody (Invitrogen, Roche, Germany) at a dilution of 1:2000. Detection was performed on an ECL-Western blotting detection system (GE Healthcare, Germany) according to the manufacturer’s protocol.

### Alignment and phylogenetic tree of galectins

The alignment of amino acid sequences was done using the online tool ‘**M**ultiple **A**lignment using **F**ast **F**ourier **T**ransform’ (MAFFT) from the EMBL-EBI, which was then processed via ‘Box shade’, an online tool maintained by the Swiss Institute of Bioinformatics (SIB). The phylogenetic tree (Fig. 3) was calculated using the program ‘MrBayes’, which uses Bayesian Inference and builds a consensus tree from millions of generations. The phylogenetic consensus tree was then processed in ‘Treegraph2’.

### Hemagglutination activity

Tandem repeat galectins with two CRDs tightly bind to N-acetyl-lactosamine determinants on blood group B erythrocytes. Lactose represents a further ligand for galectins and at high concentration detaches or prevents the interaction between galectin and blood group A determinant [38]. The hemagglutination assay was carried out according to the method described by Wang et al. [67] and Ditgen et al. [26]. A peripheral blood sample was collected from a healthy human type B blood donor in a heparinized tube. The erythrocytes were pelleted, washed three times in PBS (196 **x g** 10 min), and resuspended in PBS at a concentration of 2% (v/v). The assay was carried out in a 96-well conical microtiter plate using 10 μg recombinant *O. ochengi* galectin serially diluted triple-fold in a volume of 25 μl in PBS. To each well 25 μl of 2% erythrocyte suspension was added. After 1 h of incubation at room temperature, each well was examined visually. For inhibition of hemagglutination activity 30 μl of 200 mM lactose were pre-incubated for 30 min at room temperature with 30 μl of 2% erythrocytes suspension. Bovine serum albumin (BSA) was used as a non-agglutinating negative control protein. Afterwards, serially diluted samples were added and incubated for 1 h.

### Preparation of *O. ochengi and O. volvulus* somatic extracts

The extract preparation was done according to the methods of Brattig et al. [56] and Manchang et al. [43] with some modifications. Frozen adult worms were thawed and about 5 females ground using a mortar with 2 ml of PBS. After repeated freezing and thawing (three- times), the mixture was sonicated on ice and centrifuged at 10.000 x g. The supernatant was collected and the concentration of protein evaluated using the Bradford quantification method [68].

### Recognition of *O. ochengi* galectin by sera of immunized rats

Wistar rats (*Rattus norvegicus*) were bought from the Charles River Laboratory (Research Models and Services, Sulzfeld, Germany). The handling and immunization experiment were approved by and conducted in accordance with guidelines of the appropriate Animal Protection Board of the State of Hamburg (permit 89/09 Amt für Gesundheit und Verbraucherschutz). The Wistar rats were housed singly in ventilated steel cages under pathogen-free conditions with food and water available ad libitum. Blood samples were collected before and after immunization. After the experiment, rats were euthanized individually in a satisfied CO_2_ atmosphere of 40% of the chamber volume per minute which produces rapid unconsciousness with minimal distress (Guidelines for Euthanasia of Rodents Using Carbon Dioxide, AVMA).

Three 10 weeks old Wistar rats were each immunized with 20 μg of *O. ochengi* recombinant galectin protein through the intraperitoneal route with a 21 gauge needle syringe (BD Microlance England) and maintained in laboratory rat cages. The rats were randomly chosen from different parental lineages and similarly attributed to the *O. ochengi* galectin immunized and the mock-treated (non-immunized) groups. Blood samples were collected before immunization at day 0 (D 0), 14 days after immunization (D 14) and 14 days after boosting (D 28) centrifuged at 110 **x g**; the sera was collected and stored at −20 °C. As control, one rat was injected only with phosphate buffer solution (PBS).

The production of IgG reactive antibodies with the vaccine proteins indicated that the immune system of the mammal is activated which can block the physiological functionality of galectin, i.e. the binding to host’s cell N-acetyl-lactosamine determinants with subsequent agglutination or blocking of their physiological function. Thus, the detection of *Onchocerca* galectin-reactive IgG antibody in sera from *Onchocerca*-infected hosts – rats, cattle, humans – indicate the reactivity of the host immune cell products which may be operative in defence mechanism. The IgG binding with galectin is analysed by enzyme linked immunosorbent assay (ELISA).

The sera were analyzed by ELISA [15] for total IgG antibodies reacting with recombinant *O. ochengi* galectin and *O. ochengi* somatic extracted protein as antigens. Polystyrene microtiter plates (Maxi-Sorb, Nunc) were coated with *O. ochengi* galectin and *O. ochengi* somatic extract at a concentration of 200 ng/well in carbonate buffer (pH 9.6), sealed with Saran wrap and incubated overnight at 4 °C. After removal of unbound protein by washing three-times with PBS/0.05% (v/v) Tween 20, plates were blocked with 5% (w/v) bovine serum albumin (BSA) in PBS for 1 h at 37 °C. One hundred microliters of different dilutions (1:2000, 1:4000 and 1:8000) of rat sera were prepared in PBS/0.5% BSA, added to each well and incubated at 37 °C for 1 h. Non-specifically bound proteins were removed after washing the wells three times with PBS/0.05% (v/v) Tween 20. Detection of bound rat IgG antibodies was done using horseradish peroxidase-conjugated goat-anti-rat IgG (Dianova, Hamburg, Germany) applied at a final concentration of 1: 5000. One hundred microliters of tetramethylbenzidine (TMB) substrate (BD Biosciences, Europe) was added per well and incubated for 10 min at room temperature. The reaction was stopped with 100 μl/well of 2 M H_2_SO_4_ and the optical density (OD) determined at 450 nm (versus 620 nm) using an ELISA reader (DYNEX, Magellen Biosciences MRXII).

### Reactivity of *O. ochengi* and *O. volvulus* galectins with sera from *O. ochengi-*infected cattle and *O. volvulus*-infected humans

Analysis of serum IgG antibody levels was performed by ELISA as described previously by Mpagi et al. [55] with modifications to fit analysis with cattle sera. ELISA microtiter plates (Nunc, Roskilde, Denmark) were coated with 100 μl of 200 ng/well *O. ochengi* or *O. volvulus* galectins, and *O. ochengi* and *O. volvulus* somatic extracts were used as positive controls. After overnight incubation, plates were washed four-times with PBS 0.05% Tween 20 (pH 7.2). Excess reactive sites were blocked for 2 h at room temperature with 200 μl of PBS/5% BSA for human sera and with PBS/5% (w/v) skimmed milk for cattle plasma analyses. Furthermore, individual human sera (*n* = 44: 35 males and 9 females) from over 10 years *O. volvulus*-infected individuals diluted at 1:1000, 1:3000, and 1:9000 or sera from 2 years old *O. ochengi* infected cattle (*n* = 9: four males and five females) diluted at 1:50, 1:100, and 1:200 were used. Sera from two European cattle not exposed to *Onchocerca* spp. (EC) from the University of Veterinary Medicine (Hannover, Germany) and healthy Europeans (naïve, *n* = 12: four males and eight females) individuals were included as negative controls. The secondary antibodies used were, horseradish peroxidase-conjugated goat anti-human IgG and goat anti-bovine IgG (Sigma, St. Louis, USA), respectively, each diluted at 1:5000. Plates were processed as described above.

### Proliferative response of peripheral blood mononuclear cells (PBMCs) to *O. ochengi* galectin and *O. ochengi* extract

#### Isolation of PBMCs

About 15 to 20 ml of blood from cattle was collected in heparinized tubes and centrifuged at 784 **x g** for 15 min at room temperature. The buffy coat layer was transferred into another tube and diluted with 1x PBS (pH 7.4) at 1:2. The diluted buffy coat was cautiously overlaid onto 4 ml of Ficoll solution and then centrifuged at 544 **x g** for 45 min in order to separate the plasma, lymphocytes and erythrocytes. The lymphocyte layer was carefully removed, washed by diluting at 1:3 in ice cold 1x PBS (pH 7.4) and centrifuged at 223 **x g** for 20 min at 4 °C. The supernatant was discarded and the cells washed again with RPMI-1640 cell culture medium (500 ml RPMI-1640, 10% FCS, 200 mM L-glutamine, 2.5 ml gentamycin, 500 μl mercaptoethanol and 10 ml HEPES), by centrifuging at 223 **x g** for 10 min at 4 °C and re-suspended in fresh RPMI culture medium and the number of cells per ml determined with the aid of a Neubauer chamber.

#### PBMCs culture and cell proliferation tetrazolium dye (MTT) assay

The reactivity of immune cells of the hosts to the galectin protein or galectin in extracted proteins of *O. ochengi* stages indicates the responsiveness of the host immune system. PBMCs comprise the mononuclear immune lymphocytes (T and B cells) and monocytes. The detection of PBMC proliferation demonstrates the activation of the host’s immune system which can block the reactivity of galectin released by the parasite *O. ochengi.*

The cell proliferation was analysed by the colorimetric assay assessing the reduction of the tetrazolium dye 3-(4,5-dimethylthiazol-2-yl)-2,5-diphenyltetrazolium bromide (MTT) to formazan [69].

PBMCs were cultured in 96-well culture flat bottomed plates at concentrations of 2x10^6^cells/ml in a volume of 200 μl in RPMI-1640 cell culture medium. Cells were stimulated with recombinant *O. ochengi* galectin protein, *O. ochengi* somatic extract and polyclonal stimulant Concanavalin (ConA; Sigma-Aldrich, Germany) as a positive control at concentrations of 10 μg/ml per well. A negative control was made up of unstimulated PBMCs in culture medium only. All cultures were performed in duplicates. Cultures were incubated at 37 °C in 5% CO_2_ for 72 h after which 150 μl of the culture medium was carefully removed and the remaining 50 μl of culture medium containing cells used for the cell proliferation tetrazolium dye MTT assay (Sigma-Aldrich, Germany). Two milligrams of MTT were dissolved into 1 ml of PBS (pH 7.4), with 50 μl added onto each well containing cells and incubated at 37 °C for 3 h. Then 100 μl of sodium dodecyl sulphate SDS-dimethylformamide (DMF) (100 ml 30% SDS complemented by 50 ml DMF and 3.8 ml acetic acid) was added per well to stop the reaction. Plates were incubated in the dark over night at room temperature and the absorbance measured at 570 nm using an ELISA reader (Opsys MR, DYNEX England).

### Statistical analysis

OD values were transformed into index values (endpoint titer). This was done using a linear regression analysis as cut off with the negative control sera at an OD of 0.150 according to the method of Miura et al. [70] and Boursou et al. [58]. Graphs were drawn using GraphPad prism 6. SSPS 16.0 (Chicago, Illinois, USA) was used for statistical analysis using the nonparametric Mann-Whitney *U* test for significant differences in cell proliferation between the *O. ochengi* low and highly infected cattle. The median and interquartile range (Med: 25–75%) were used; differences were considered statistically significant when *p*< 0.05.

## Supplementary Information

**Additional file 1. **Reactivity and cross-reactivity of IgG in sera from *Onchocerca*-infected cattle and humans with *O. ochengi* and *O. volvulus* galectins and extracted proteins.

## Data Availability

All data generated or analyzed during this study are included in this published article and its supplementary information files are available from the corresponding author on reasonable request.

## Abbreviations

BSA: Bovine serum albumin
CRD: Carbohydrate recognition domain
ESP: Excretory- secretory products
ELISA: Enzyme-linked immuno-sorbent assay
EC: European cattle, Immunoglobulin G
IQR: Interquartile range
Mff: Microfilaria
PBMC: Peripheral blood mononuclear cell
PBS: Phosphate buffered saline
PCR: Polymerase chain reaction
SDS-PAGE: Sodium dodecyl sulphate polyacrylamide gel electrophoresis
TCA: Trichloroacetic acid
TMB: Tetramethylbenzidine
