# Correction to: Galectins from Onchocerca ochengi and O. volvulus and their immune recognition by Wistar rats, Gudali zebu cattle and human hosts

**DOI:** 10.1186/s12866-021-02177-3

**Published:** 2021-04-19

**Authors:** Ngwafu Nancy Ngwasiri, Norbert W. Brattig, Dieudonné Ndjonka, Eva Liebau, Archile Paguem, Dustin Leusder, Manchang Tanyi Kingsley, Albert Eisenbarth, Alfons Renz, Achukwi Mbunkah Daniel

**Affiliations:** 1 University of Ngaoundéré, Ngaoundéré, Cameroon; 2 Department Molecular Medicine, Bernhard Nocht Institute of Tropical Medicine, Hamburg, Germany; 3 University of Muenster, Münster, Germany; 4 Department Comparative Zoology, Eberhard Karls University, Institute of Evolution and Ecology, Tübingen, Germany; 5 Department of Veterinary Medicine, University of Buea, Buea, Cameroon; 6 Veterinary Research Laboratory, IRAD Wakwa Regional Centre, Ngaoundéré, Cameroon; 7 Programme Onchocercoses, Station of the University of Tübingen, Ngaoundéré, Cameroon; 8 TOZARD Research Laboratory, P.O. Box 59, Bambili-Tubah, Bamenda, Cameroon

**Correction to: BMC Microbiol 21, 5 (2021)**

**https://doi.org/10.1186/s12866-020-02064-3**

Following the publication of the original article [1], we were notified that an incorrect text had been published in the Competing interests section. This has been removed.

The original article has been corrected.
