# Supplementary material for: Galectins from Onchocerca ochengi and O. volvulus and their immune recognition by Wistar rats, Gudali zebu cattle and human hosts
Source: BMC Microbiol. 2021 Jan 6;21:5. doi: 10.1186/s12866-020-02064-3 (PMC7788699; doi:10.1186/s12866-020-02064-3)
Supplement: Supplementary file 1 — Additional file 1. Reactivity and cross-reactivity of IgG in sera from Onchocerca-infected cattle and humans with O. ochengi and O. volvulus galectins and extracted proteins. [file 12866_2020_2064_MOESM1_ESM.doc]

| **Additional file 1.** Reactivity and cross-reactivity of IgG in sera from *Onchocerca*-infected cattle and humans with *O. ochengi* and *O. volvulus* galectins and extracted proteins | | | | | |
| --- | --- | --- | --- | --- | --- |
| **Antigens** | **OD of sera from European control cattle** | **OD of sera from healthy European humans** |  | **OD of sera from**  ***O. volvulus-*infected**  **human patients** | **OD of sera from**  ***O. ochengi-*infected**  **cattle** |
|  |  | **Median (IQR)** |  | **Median (IQR)** | **Median (IQR)** |
| *O. ochengi* galectin | 0 | 0(0-126) |  | 5353 (307-6211) | 0.0 (0.0-164.0) |
| *O. volvulus* galectin | 0 | 194(0-311) |  | 5787 (4969-6217) | 192 (71-224) |
| *O. ochengi* extract | 0 | 0(0-129) |  | 11214 (9408-13576) | 332 (299-371) |
| *O. volvulus* extract | 0 | 182(0-294) |  | 12863 (10772-18164) | 370 (327-507) |
| IgG reactivity of *O. ochengi* galectin*, O. volvulus* galectin and crude somatic extract (extracted proteins) with sera from *O. ochengi-*infected cattle (n = 9), *O. volvulus-*infected humans (n = 44) naïve European cattle (n = 2), and healthy Europeans (n = 12). OD: Optical density; IQR: interquartile range of OD values enclosed in brackets. No OD values were observed with the European control cattle using all antigens. On the other hand, very low signals (OD) were observed in some European humans with the various antigens**.** | | | | | |
